# Biochemical and Structural Characterization of a Novel Bacterial Tannase From *Lachnospiraceae bacterium* in Ruminant Gastrointestinal Tract

**DOI:** 10.3389/fbioe.2021.806788

**Published:** 2021-12-15

**Authors:** Lijun Guan, Kunlun Wang, Yang Gao, Jialei Li, Song Yan, Nina Ji, Chuanying Ren, Jiayou Wang, Ye Zhou, Bo Li, Shuwen Lu

**Affiliations:** ^1^ Institute of Food Processing, Heilongjiang Academy of Agricultural Sciences, Harbin, China; ^2^ Heilongjiang Province Key Laboratory of Food Processing, Harbin, China; ^3^ Soybean Institute, Heilongjiang Academy of Agricultural Sciences, Harbin, China; ^4^ Biotechnology Research Institute, Heilongjiang Academy of Agricultural Sciences, Harbin, China

**Keywords:** bacterial tannase, biochemical characterization, kinetic analysis, homology modeling, structural analysis

## Abstract

Tannases are a family of esterases that catalyze the hydrolysis of ester and depside bonds present in hydrolyzable tannins to release gallic acid. Here, a novel tannase from *Lachnospiraceae bacterium* (TanA_Lb_) was characterized. The recombinant TanA_Lb_ exhibited maximal activity at pH 7.0 and 50°C, and it maintained more than 70% relative activity from 30°C to 55°C. The activity of TanA_Lb_ was enhanced by Mg^2+^ and Ca^2+^, and was dramatically reduced by Cu^2+^ and Mn^2+^. TanA_Lb_ is capable of degrading esters of phenolic acids with long-chain alcohols, such as lauryl gallate as well as tannic acid. The *K*m value and catalytic efficiency (*k*_cat_ /*K*m) of TanA_Lb_ toward five substrates showed that tannic acid (TA) was the favorite substrate. Homology modeling and structural analysis indicated that TanA_Lb_ contains an insertion loop (residues 341–450). Based on the moleculer docking and molecular dynamics (MD) simulation, this loop was observed as a flap-like lid to interact with bulk substrates such as tannic acid. TanA_Lb_ is a novel bacterial tannase, and the characteristics of this enzyme make it potentially interesting for industrial use.

## Introduction

Tannins are water-soluble polyphenolic compounds that constitute the fourth most abundant class of plant biomass components, richly secreted by defensive systems in wood, fruits, roots, and seeds (White, 1957; Smeriglio et al., 2016; Maisetta et al., 2019). Tannins and their derivatives are the key compounds of the astringent taste of tea, fruits, and vegetables, and determine the quality of their production such as muddy and astringency problem of juice and beer (Charlton et al., 2002). In addition, tannins inhibit the growth of microbes, exert antinutritional effect on animals and are even toxic to the human body in that they possess the capacity to bind and precipitate proteins and to bind iron, which could lead to many biological process disorders (Mcdonald et al., 1996; Baxter et al., 1997; Chung et al., 1998; Aguilar et al., 2007; Smeriglio et al., 2016). During millions of years of biological evolution, various microorganisms have adapted to utilize tannins as carbon and/or energy sources for growth and development using enzymes such as tannin acyl hydrolases, generally known as tannases (Lekha and Lonsane, 1997; Aguilar et al., 2007). Tannases (EC 3.1.1.20) catalyze the hydrolysis of ester and depside bonds present in gallotannins, complex tannins, and gallic acid esters, resulting in the release of glucose and gallic acid (Govindarajan et al., 2016). Tannase have broad practical applications in the food, feed, beverage, pharmaceutical, agricultural, and the leather industries. Compared with chemical hydrolysis methods catalyzed by HCl, nucleophiles and precious metals, the bio-hydrolysis of tannins has unique advantages due to its low environmental impact (Aharwar and Parihar, 2018). Thus, there is an increasing demand for tannases with desirable biochemical properties in industry.

Most tannases that are biochemically studied and characterized in detail come from fungi (Aboubakr et al., 2013), but not much is known about tannase of bacteria or yeasts living under extreme conditions, which are considered to be the potential of tannases with unexpected characteristics (Dhiman et al., 2018; Tomás-Cortázar, 2018). The studied tannases in bacteria are almost from the rumen, gut, oral cavity microbiota, or soils (Noguchi et al., 2007; Wu et al., 2014; Chaitanyakumar and Anbalagan, 2016; Tomás-Cortázar et al., 2018). Recently, it is reported that plant tannase was first identified from leaves of tea, strawberry, and crops, which hydrolyze tannin compounds, glycosides, and ph enolic glycosides to produce toxic small molecule phenolic compounds, so as to protect plants from attack by microorganisms and herbivore (Dai et al., 2020).

Tannase includes extracellular tannases encoded by the tanA gene and intracellular tannases encoded by tanB gene, with molecular size of 66 and 50 kDa, respectively (Tomás-Cortázar et al., 2018). Exceptionally, TanB_SS_ encoded by a tanB gene from *Streptomyces sviceus* was identified as an extracellular tannase (Wu et al., 2014). The optimum temperature and pH value for biochemical characteristics of bacterial tannases are 30°C–50°C and 3.0–7.0, respectively (Lekshmi et al., 2021). Up to now, two kinds of bacterial tannase belonging to probiotics (TanA_Lp_, *Lactobacillus plantarum*) and the oral bacteria (TanB_Fnn_, *Fusobacterium nucleatum*) have been structurally characterized by combining crystal structure, molecular dynamics, and mutation analysis, which confirmed the functional role of the flap domain and flap-like domain in the substrate recognition and specificity of tannase (Ren et al., 2013; Mancheño et al., 2020).

Here, a putative bacterial tannase from a species of *Lachnospiraceae bacterium* was characterized in detail. Recombinant TanA_Lb_ was cloned, overexpressed, and purified in order to fully analyze the biochemical characteristics. To understand the biochemical characteristics of TanA_Lb_, three-dimensional structure of the enzyme was modeled. Assisted by molecular docking and MD simulation, the structure-oriented substrate specificity analysis was carried out. This work adds a new bacterial tannase with comprehensive biochemical and structural characterization to provide a platform for the manipulation of tannins in food and beverage products, or for transformation into biologically active products to benefit human health on the basis of specific requirements.

## Materials and Methods

### Cloning and Expression of Tannase From *Lachnospiraceae Bacterium*

The coding sequence of TanA_Lb_ (GenBank accession No. MBQ6323131.1) was codon optimized for *E*. *coli* and synthesized by Genewiz (Beijing, China). The purified PCR products were subcloned into the pET-28a (+) vector (Novagen, Madison, WI, USA) between the *Nde* I and *EcoR* I restriction sites, resulting in the addition of a 6×His tag at the N-terminus. The primer sequences are TanA_Lb__F (GGA​ATT​CCA​TAT​GTC​ACA​GTC​AAC​AGC​TAC​ACG​C) and TanA_Lb__R (GGA​ATT​CAA​GGC​CAG​CAG​CTT​TAA​GGC​G). *E. coli* BL21 (DE3) was transformed with the resulting expression vector pET-28a-TanA_Lb_ and grown in Luria–Bertani (LB) broth medium with 50 µg/ml kanamycin overnight at 37°C. On the next day, a fresh culture in the same medium was inoculated, and when it reached 0.6–0.8, isopropyl β-D-1-thiogalactopyranoside (IPTG) was added to a final concentration of 0.5 mM, to induce the expression of pET-28a-TanA_Lb_ at 16°C overnight. The cells were harvested by centrifugation for 15 min at 5,000 × g and 4°C, and washed with 0.9% NaCl solution.

### Purification of the Recombinant Tannase From *Lachnospiraceae bacterium*

The cells expressing recombinant TanA_Lb_ were resuspended in lysis buffer [20 mM Tris-HCl, 20 mM imidazole, 500 mM NaCl, 1 mM dithiothreitol (DTT), pH 8.0] and ultrasonicated at 1.5-s pulse and 3-s output for 15 min. Then, the crude lysate was centrifuged at 40,000 × *g* and 4°C for 30 min, and the supernatant was bound to a 1 ml of Ni-NTA Superflow column (Qiagen, Hilden, Germany). After washing with 10 ml of wash buffer (20 mM Tris-HCl, 0.5 M NaCl, 1 mM DTT, 50 mM imidazole, pH 8.0), the TanA_Lb_ protein was eluted with 10 ml of elution buffer (20 mM Tris-HCl, 0.3 M NaCl, 1 mM DTT, 0.5 M imidazole, pH 8.0). For further purification, the target protein was buffer exchanged into 20 mM Tris–HCl pH 8.0, 1 mM DTT, loaded onto a 5-ml MonoQ ion exchange column (GE Healthcare, Piscataway, NJ, USA), and eluted using a linear gradient using from 0 to 1 M NaCl. Then, gel filtration chromatography was used to determine the apparent mass of the TanA_Lb_ protein using a Superdex 200 increase column (GE Healthcare, Piscataway, NJ, USA). The running buffer was 20 mM Tris-HCl (pH 8.0, containing 150 mM NaCl and 1 mM DTT), and the flow rate was 0.5 ml/min. Finally, the purified protein was stored at 4°C until further analysis. The molecular mass and purity of the target protein were assessed by SDS-PAGE.

### Enzyme Activity Assay

The enzyme activity of TanA_Lb_ was determined using the rhodamine assay published by Inoue and Hagerman (Jiménez et al., 2014). TanA_Lb_ (100 μg) in 700 μl of 50 mM phosphate buffer, pH 6.5, was incubated with 40 μl of 25 mM methyl gallate (final substrate concentration 1 mM), for 5 min at 37°C. After incubation, 150 μl of 0.667% rhodamine in 100% methanol was added to the mixture. After incubation at 30°C for 5 min, 100 μl of 0.5 M KOH was added and incubated for an additional 5–10 min, after which the absorbance was measured at 520 nm using a spectrophotometer. A standard curve was prepared using gallic acid solutions ranging from 0.125 to 1 mM. One unit of TanA_Lb_ activity was defined as the amount of enzyme that releases 1 μM gallic acid per minute under the described assay conditions. The concentration of TanA_Lb_ was determined using a BCA Protein Assay Kit (Solarbio, China).

To determine the substrate specificity of TanA_Lb_, 1 mM methyl gallate (MG), ethyl gallate (EG), propyl gallate (PG), lauryl gallate (LG) and tannic acid (TA) were used as the substrate, and enzyme activity was measured at 50°C and pH 7.0. The kinetic parameters of the purified enzymes were determined by assaying the activity on various substrate concentrations. MG, EG, PG, LG (0.2–5 mM) and tannic acid (0.01–0.05 mM) were incubated with appropriate amount of enzyme to calculate the kinetics. The amount of gallic acid which was formed by the catalysis of tannase was calibrated using the absorbance at 520 nm. Kinetic parameters were obtained according to the Line-weaver and Burk method. Each measurement was conducted three times. All calculations were performed using the Origin 9.0 software.

### Effects of Temperature, pH, and Additives on the Activity and Stability of the Enzyme

The optimal temperature of TanA_Lb_ was determined by measuring the relative activities in the range of 20°C–70°C in 20 mM phosphate buffer saline at pH 7.0. To evaluate the thermal stability of TanA_Lb_, the residual activity was measured after pre-incubation in 20 mM phosphate buffer saline buffer (pH 7.0) at 20°C, 30°C, 40°C, 50°C, 60°C, and 70°C, respectively. Samples were taken every 30 min for 5 h.

Different buffers were used to assess the optimal pH of TanA_Lb_ at 50°C, with methyl gallate as the substrate. The pH value was varied from 2.5 to 10 using 20 mM glycine-HCl buffer (pH 2.5–5.5), 20 mM 4-morpholineethanesulfonic acid (MES) (pH 5.5–6.5), 20 mM phosphate buffer saline (pH 6.5–8.0), 20 mM Tris-HCl (pH 8.0–9.5), and 20 mM glycine-NaOH (pH 9.5–10.0). In order to determine the pH stability of TanA_Lb_, the enzyme was incubated in the indicated buffers (pH 2.5–10.0) at 50°C for 30 min, and the residual activity was measured under the standard conditions.

The effects of various metal ions on the purified recombinant TanA_Lb_ were investigated by measuring the relative enzyme activity in the presence of 1 mM K^+^, Ca^2+^, Co^2+^, Mn^2+^, Mg^2+^, Zn^2+^, and Cu^2+^, respectively. The enzyme activity was measured at pH 7.0 and 50°C. Additionally, the enzyme was incubated in 20 mM phosphate saline buffer pH 7.0 with 1 mM ethylene diamine tetraacetic acid (EDTA), sodium dodecyl sulfate (SDS), Tween-80, dimethyl sulfoxide (DMSO), and TritonX-100 at 25°C for 1.0 h to investigate the effect of a chelating agent. The activity without adding any additives was defined as 100%.

### Homology Modeling of Tannase From *Lachnospiraceae bacterium*

Modeller 9.9 software (http://salilab.org/modeller/) was used to generate a homology model of TanA_Lb_ (Gao et al., 2012). The crystal structure of a homologous protein from *Fusobacterium nucleatum* subsp. *nucleatum* (PDB ID: 6YQ4), which has 42.9% sequence identity with the target protein TanA_Lb_, was chosen as template. A sequence alignment between TanB_Fnn_ and TanA_Lb_ was generated automatically using the align2d command. Subsequently, homology modeling was executed using the auto-model command. The best model was chosen based on the MODELLER objective function values and the DOPE assessment scores. Quality of the predicted 3D structural model was assessed by the PROCHECK (https://servicesn. mbi.ucla.edu/PROCHECK). The constructed model structure was visualized and analyzed using PyMOL software (http://www.pymol.org).

### Molecular Docking

MOE package (Molecular Operating Environment, 1010 Sherbooke St. West, Suite #910, Montreal, QC, Canada, H3A 2R7. 2018) was used for molecular docking of TanA_Lb_ with five ligands and predicting the binding affinity, respectively. The 2D structure of ligands were downloaded from the PubChem web site (http://pubchem.ncbi.nlm.nih.gov) and converted to 3D in MOE through energy minimization. Then the protonation state of target and the orientation of the hydrogens were optimized by LigX, at the PH of 7 and temperature of 300 K. Prior to docking, the force field of AMBER10:EHT and the implicit solvation model of Reaction Field (*R*-field) were selected (Case et al., 2008). The position of the native ligand (SPD) in the X-ray structure of 6YQ4 which is selected as the homology template of TanA_Lb_ was defined as the binding site. The docking workflow followed the “induced fit” protocol, in which the side chains of the receptor pocket were allowed to move according to ligand conformations, with a constraint on their positions. The weight used for tethering side chain atoms to their original positions was 10. All docked poses of molecules were ranked by London dG scoring first, and then a force field refinement was carried out on the top 30 poses followed by a rescoring of GBVI/WSA dG.

### Molecular Dynamics (MD) Simulation

For the complex structure of TanA_Lb_ and tannic acid, whose structure was neutralized by adding sodium/chlorine counter ions and solvated in a cuboid box of TIP3P water molecules with solvent layers 12 Å between the box edges and solute surface.

All MD simulations were performed using AMBER20 (Case et al., 2021). The AMBER FF19SB and gaff2 force fields were applied, and the SHAKE algorithm was used to restrict all covalent bonds involving hydrogen atoms with a time step of 2 fs. The Particle mesh Ewald (PME) method was employed to treat long-range electrostatic interactions. For the solvated system, two steps of minimization were performed before the heating step. The first 4,000 cycles of minimization were performed with all heavy atoms restrained with 50 kcal/(mol·Å^2^), whereas solvent molecules and hydrogen atoms were free to move. Then non-restrained minimization was carried out involving 2,000 cycles of steepest descent minimization and 2,000 cycles of conjugated gradient minimization. Afterward, the whole system was first heated from 0 to 300 K in 50 ps using Langevin dynamics at a constant volume and then equilibrated for 400 ps at a constant pressure of 1 atm. A weak constraint of 10 kcal/ (mol·Å^2^) was used to restrain all the heavy atoms during the heating steps. Periodic boundary dynamics simulations were carried out for the whole system with an NPT (constant composition, pressure, and temperature) ensemble at a constant pressure of 1 atm and 300 K in the production step. In production phase, a 10-ns simulation was carried out. The trajectories were further analyzed using Cpptraj (Roe and Cheatham., 2013). The binding free energy of complexes was calculated using the MM-GBSA method.

## Results and discussion

### Multiple Sequence Alignment and Expression of Recombinant Tannase From *Lachnospiraceae Bacterium*

**FIGURE 1:**
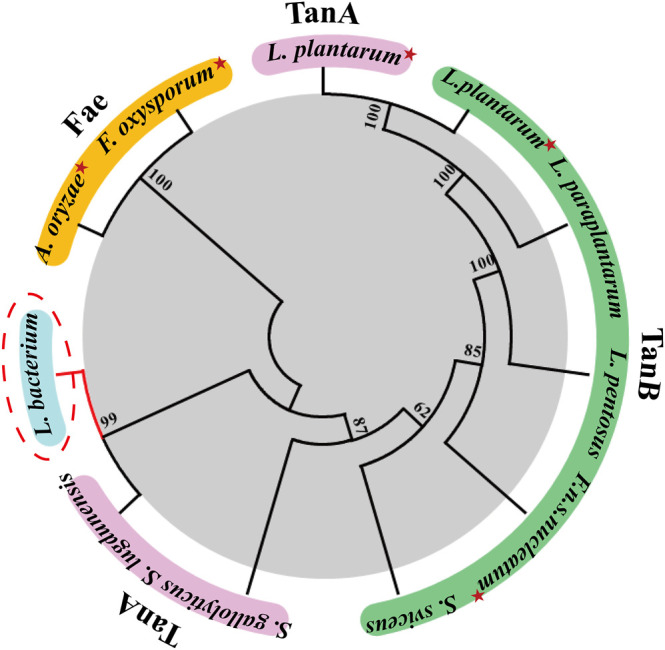
Phylogenetic tree of tannase from *Lachnosperaceae bacterium* (TanA_Lb_) and homologous enzymes. Tannases with similar amino acid sequences to TanA_Lb_ were searched in the UniProt and NCBI databases, and the phylogenetic tree was constructed using MEGA 7.0. Enzymes with solved crystal structures are indicated with red star.

A search in the NCBI database for homologues tannase sequences yielded the putative TanA_Lb_ protein from a species belonging to the family *Lachnospiraceae* (GenBank Accession No. MBQ6323131.1). A multiple sequence alignment was performed based on the protein sequence of TanA_Lb_ with characterized tannase from other organisms (Supplementary Figure S1). The sequence alignment results revealed that TanA_Lb_ showed 42.9% sequence similarity with TanB_Fnn_ from *Fusobacterium nucleatum* subsp. *nucleatum* (GenBank Accession No. ALQ42581.1) (Mancheño et al., 2020) and 40.4% with TanASl from *Staphylococcus lugdunensis* (GenBank Accession No. BAF03594.1) (Rusniok et al., 2010). However, it showed only 24% sequence similarity with TanB_Lp_ from *Lactobacillus plantarum* (GenBank Accession No. AB379685.1) (Matoba et al., 2013). TanA_Lb_ exhibited the conserved catalytic triad (Ser205, His614, Asp582) typical for the tannase family (Supplementary Figure S1). Furthermore, TanA_Lb_ also contained the conserved motif Gly203-X-Ser205-X-Gly207, which is typical of serine hydrolases (Ren et al., 2013). A phylogenetic analysis of TanA_Lb_ and previously identified tannases indicated that TanA_Lb_ is more similar with TanA_Sl_ than the other investigated enzymes (Figure 1). Multiple sequence alignment and phylogenetic analysis, therefore, both indicated that TanA_Lb_ may have tannase activity.

**FIGURE 2:**
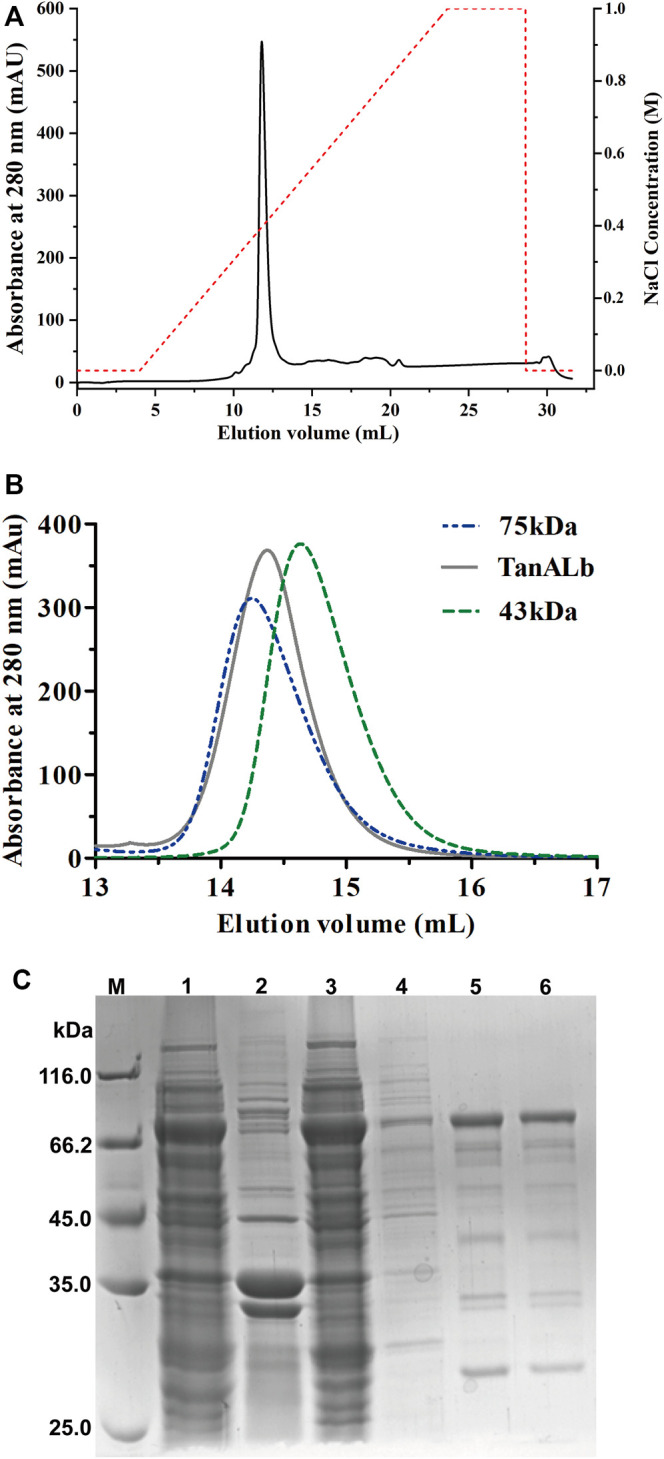
Purification of recombinant TanA_Lb_ protein. **(A)** The purification of TanA_Lb_ by ion exchange chromatography. **(B)** The purification of TanA_Lb_ by size-exclusion chromatography. **(C)** SDS-PAGE analysis of the expression and purification of His6-TanA_Lb_. *Lane M*: protein molecular weight markers; *Lanes 1–2*: crude extract; *Lane 3*: flowthrough; *Lane 4*: unbound proteins; *Lane 5*: the sample before elution; *Lane 6*: fractions eluted from his affinity resin.

TanA_Lb_ was purified using Ni-NTA affinity chromatography, ion exchange (Figure 2A) and size-exclusion chromatography (Figure 2B) of which the purification multiple was probably 6.9. The purified TanA_Lb_ was analyzed *via* SDS-PAGE and showed a single protein band with an apparent molecular weight of approximately 68 kDa (Figure 2C), which was in agreement with the predicted molecular weight. Furthermore, size-exclusion chromatography of purified TanA_Lb_ was performed to determine its native state in solution. A single peak was observed between the peaks of the standard protein markers actin from rabbit muscle (43 kDa) and conalbumin (75 kDa), which indicated that TanA_Lb_ was a monomer in solution (Figure 2B).

### Biochemical Characterization of Tannase From *Lachnospiraceae Bacterium*

Since tannic acid is almost exclusively formed from poly-galloyl glucose derivatives, it was used as substrate to assess if TanA_Lb_ can catalyze the hydrolysis of natural tannin. The gallic acid resulting from the degradation was identified using the rhodamine method, which indicated that tannic acid was hydrolyzed by TanA_Lb_.

**FIGURE 3:**
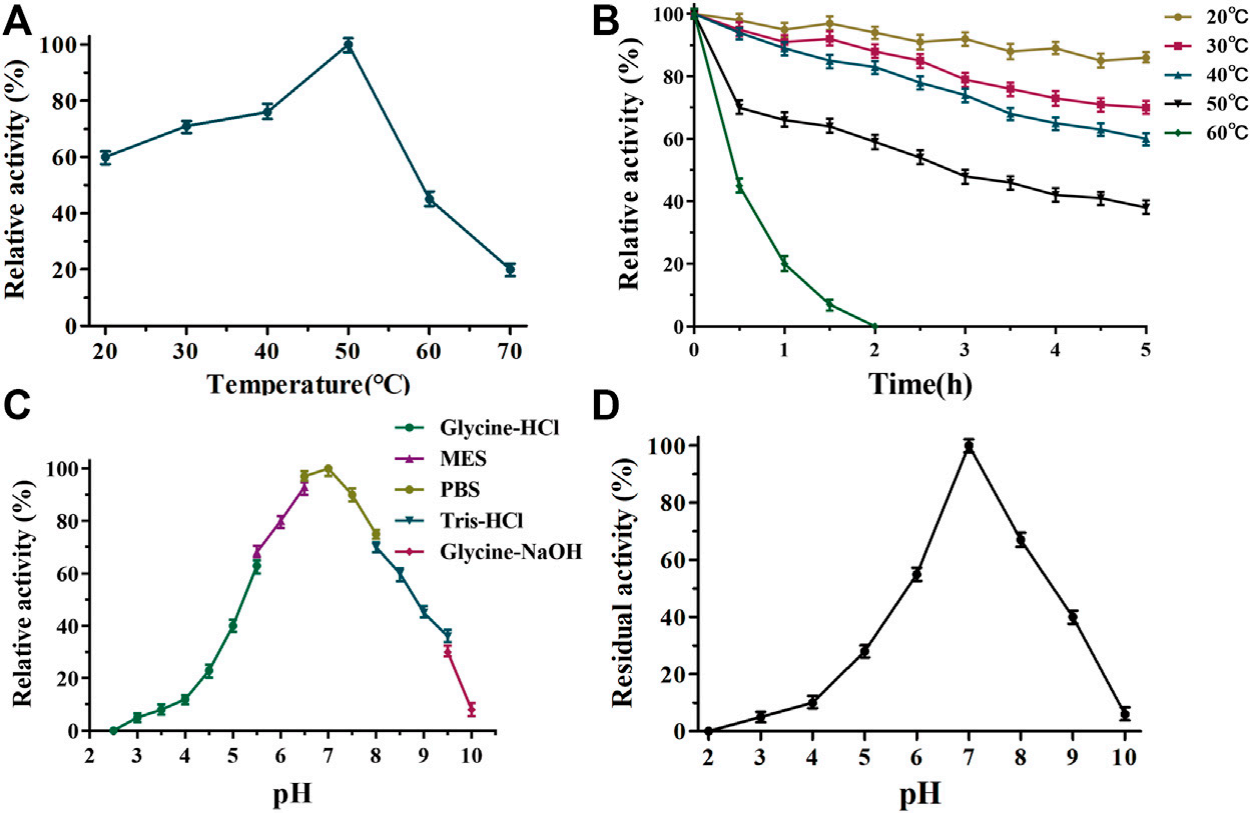
Determination of the optimal temperature **(A)**, thermostability **(B)**, pH optimum **(C),** and pH stability **(D)** of TanA_Lb_.

The biochemical properties of TanA_Lb_ were characterized using methyl gallate (MG) as the substrate. The temperature–activity profile revealed that the purified TanA_Lb_ displayed the highest activity at 50°C, which was determined by incubating the TanA_Lb_ in 20 mM phosphate buffer saline at pH 7.0. TanA_Lb_ maintained more than 70% relative activity in a rather wide temperature range from 30°C to 55°C. However, the activity of TanA_Lb_ decreased dramatically at temperatures over 60°C. (Figure 3A). Furthermore, the thermostability of the purified TanA_Lb_ was also measured by pre-incubation at different temperatures in the range of 20°C–60°C. After pre-incubation of the enzyme between 20°C and 40°C, it maintained more than 70% residual activity, which indicated that TanA_Lb_ was stable at moderate temperatures. However, incubation at temperature 60°C induced a dramatic decrease in TanA_Lb_ activity within 30 min, and the enzyme was completely inactivated after 2 h (Figure 3B). Thus, TanA_Lb_ was highly active in a wider range of temperatures, from 20°C to 50°C, exhibiting much higher thermostability than the previously reported tannases from *L. plantarum* (Curiel et al., 2009) and *B. subtilis* (Jana et al., 2013).

TanA_Lb_ exhibited measurable activity toward methyl gallate at pH 2.5–10, with optimal activity in 20 mM phosphate buffer at pH 7.0. Furthermore, it maintained more than 80% of the maximal activity at pH 5.5–7.5 (Figure 3C). TanA_Lb_ was also incubated for 2 h in different buffers to investigate its pH stability. Over 80% of the initial enzyme activity remained after incubation for 2 h at pH 6.5–7.5 (Figure 3D), while the residual activity decreased dramatically at pH values from 2–6.5 and 6.5–10.0.

The enzymatic activity of TanA_Lb_ in the presence of various additives was determined by pre-incubating the enzyme at 50°C for 1.0 h, and the effects on the activity of TanA_Lb_ are shown in Table 1. When Mg^2+^ and Ca^2+^ ions were added, the relative activity of TanA_Lb_ was enhanced by 130% and 122%, respectively. Zn^2+^ and Co^2+^ had a weak influence on the activity of TanA_Lb_, while Cu^2+^ and Mn^2+^ had a strong inhibitory effect. Additionally, the enzyme activity was increased by surfactants, such as SDS, Tween-80, DSMO, and TritonX-100, while decreased by the metal chelating agent EDTA.

**TABLE 1:** Effects of metal ions, inhibitors, and surfactants on the activity of tannase from *Lachnosperaceae bacterium* (TanA_Lb_).

		Residual activity (%) *
Metal ions	Control	100
	K^+^	72
	Ca^2+^	115
	Co^2+^	80
	Mn^2+^	51
	Mg^2+^	124
	Zn^2+^	75
	Cu^2+^	33
Surfactant and inhibitor	Control	100
	SDS	105
	DMSO	133
	Tween-80	127
	Triton X-100	122
	EDTA	78

### Kinetic Analysis on Substrate Preference of Tannase From *Lachnospiraceae bacterium*

Tannase catalyzes the hydrolysis of the galloyl ester bond, liberating gallic acid. Accordingly, tannase activity can be determined by measuring the concentration of gallic acid formed by the enzyme. In order to investigate the substrate specificity of TanA_Lb_ different esters of gallic acid, methyl gallate (MG), ethyl gallate (EG), propyl gallate (PG), lauryl gallate (LG), and tannic acid (TA) were transformed into gallic acid by TanA_Lb_ under the optimal catalytic conditions. The *K*m and *k*_cat_ value were always used to compare the substrate specificity of enzymes. *K*m values of five kinds of substrates showed that tannic acid (TA) had the highest affinity, laurate gallate (LG) had lower activity than tannic acid, and methyl gallate (MG) had the lowest affinity for the TanA_Lb_ (Table 2). The activity assay also showed that the catalytic efficiency (*k*_cat_/*K*m) of TanA_Lb_ toward tannic acid (TA) was 5.3- to 12.5-fold larger than that toward the other four substrates. Analysis of catalytic efficiency implied that TanA_Lb_ was conducive to using tannic acid (TA) as substrate. On the basis of these results, we can conclude that TanA_Lb_ has high substrate affinity and catalytic efficiency for depside bonds. In addition, TanA_Lb_ is capable of degrading not only esters of phenolic acid with short chain aliphatic alcohols but also esters of phenolic acids with long chain alcohols such as lauryl gallate, similar to TanB_Fnp_ and TanB_Fnn_ (Tomás-Cortázar et al., 2018; Mancheño et al., 2020).

**TABLE 2:** Kinetic parameters of TanA_Lb_.

Substrate	*K*_m_ (mM)	*k*_cat_ (s^−1^)	*k*_cat_ /*K*_m_ (mM^−1 s−1^)
MG	2.8 ± 0.35	82.5 ± 6.3	36.4 ± 4.4
EG	2.6 ± 0.19	78.8 ± 10.4	33.3 ± 7.3
PG	2.0 ± 0.22	44.2 ± 3.6	24.5 ± 4.0
LG	1.8 ± 0.26	24.6 ± 5.2	15.3 ± 2.1
TA	0.47 ± 0.12	82.1 ± 11.6	192.51 ± 4.3

### Molecular Structure Simulation and Structural Analysis of Tannase From *Lachnospiraceae bacterium*

**FIGURE 4:**
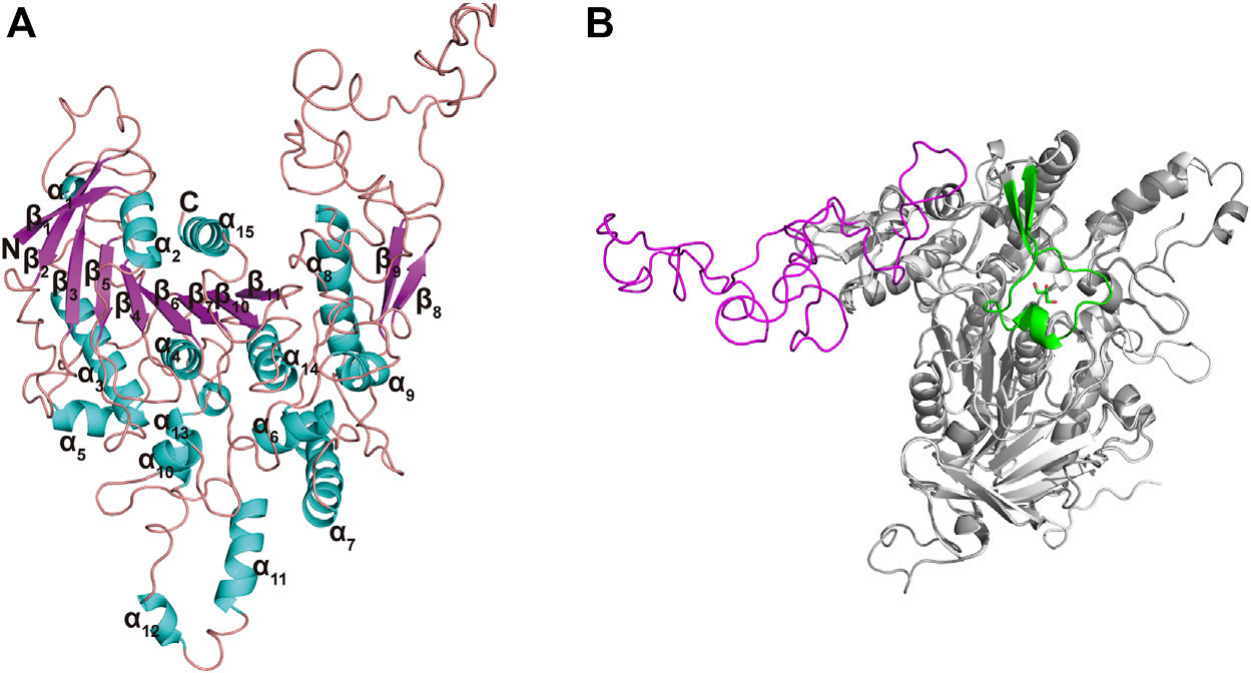
Ribbon representation of the TanA_Lb_ homology model. **(A)** The modeled 3D-structure of TanA_Lb_ based on the crystal structure of TanB_Fnn_ from *Fusobacterium nucleatum subsp. Polymorphum* (PDB code, 6YQ4). **(B)** Superimposition of the structures of TanA_Lb_ (gray, ribbon model) and TanB_Fnn_ (white, ribbon model) reveals significant differences between both proteins predominantly found in the flap region. The flap lid in TanB_Fnn_ is shown in green and the hypothetic one in TanA_Lb_ is shown in magenta.

The Ramachandran plot (Supplementary Figure S2) for TanA_Lb_ showed that 98% residues were in allowed regions, indicating that the 3D structure of the model was reasonable. The structure of TanA_Lb_ was composed of nine *β*-strands and six *α*-helices. The *β*-strands formed a twisted *β*-sheet, in which *β*2 runs antiparallel to the other *β*-strands, similar to the crystal structure of TanB_Fnn_. Furthermore, the structure of TanA_Lb_ exhibited an almost parallel six-strand *β*-sheet surrounded by five *α* helices, two of which were located on the concave side of the sheet (*α*2 and *α*15) and three on the convex side (*α*3, *α*4, and *α*14), to form a central cavity (Figure 4A). In agreement with the sequence alignment, the structural characterization, therefore, indicated that TanA_Lb_ belongs to the *α*/*β*-hydrolase superfamily (Remington et al., 1992). It is worth noting that the TanA_Lb_ contained an insertion of a 109 residue stretch (residues 341–450) (Figure 4B), which is the largest structural deviation and may assume the role of the flap covering the active site of the enzyme (Mancheño et al., 2020).

### Molecular Docking and Molecular Dynamics Simulation

**FIGURE 5:**
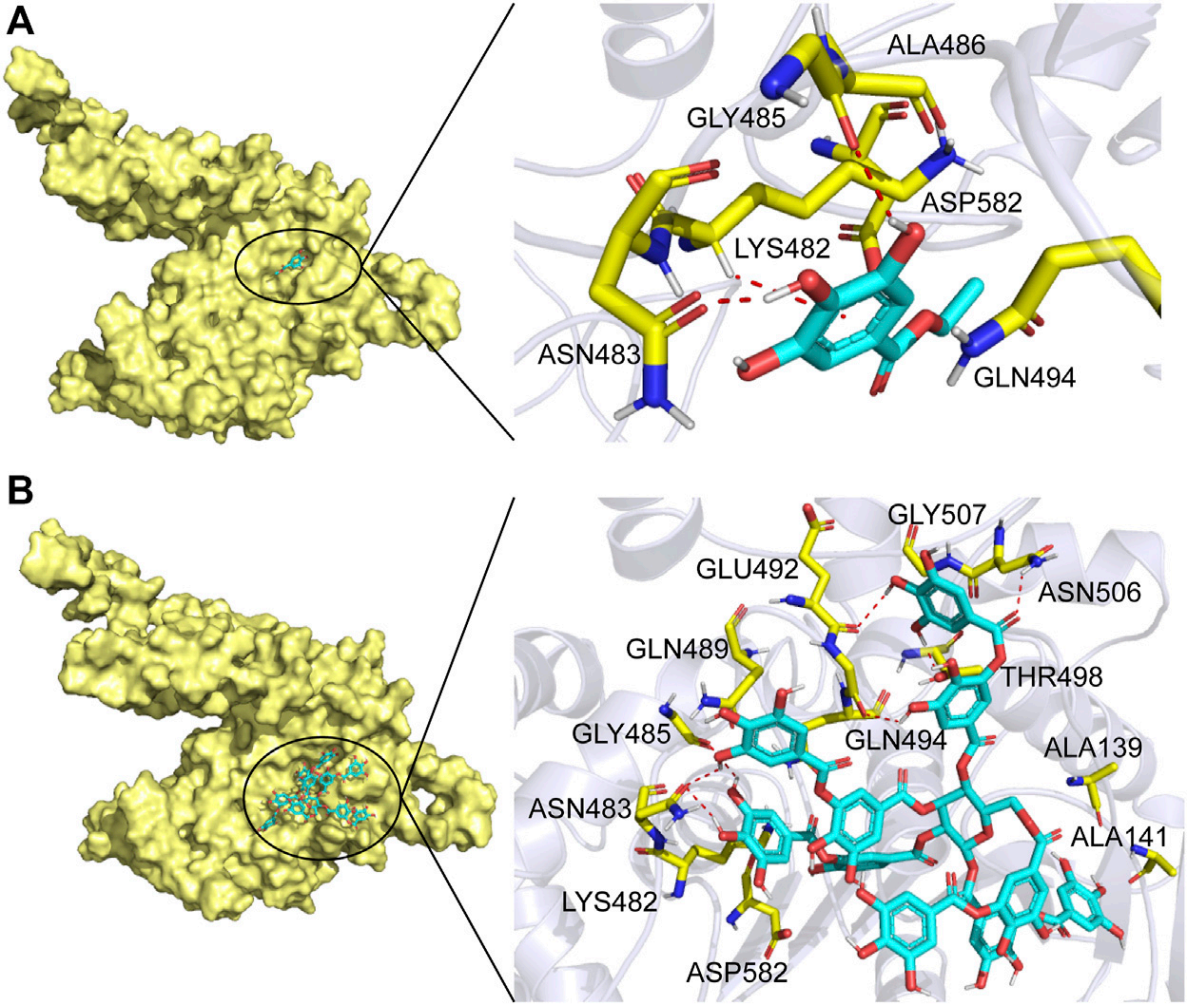
Substrate binding mode to TanA_Lb_. **(A)** The binding model of ethyl gallate on molecular surface of TanA_Lb_. **(B)** The binding model of tannic acid on molecular surface of TanA_Lb_. Substrates are colored cyan, and the molecular surface of TanA_Lb_ is colored pale yellow. The residues involved in binding are shown in yellow sticks.

In order to further understand the mechanism of action of TanA_Lb_, molecular docking and MD simulation were carried out. In the overall docking conformation of TanA_Lb_, the substrates have formed a suitable steric complementarity with the binding site of TanA_Lb_. The docking scores (Table 3), the more negative, the better binding with protein, were consistent with the kinetic analysis. The contact residues during docking of ligands on the predicted structure of TanA_Lb_ are presented in Figure 5 and Supplementary Figures S3–S5. Concretely, hydrogen bond interactions and H-π stacking were formed between TanA_Lb_ and ethyl gallate. The oxygen atom of ethyl gallate, regarded as hydrogen bond donor, forms a hydrogen bond with the oxygen atom of backbone of Gly485 and the oxygen atom of side chain of Asn483. The benzene ring of ethyl gallate, forms a CH-π stacking with the alpha carbon atom of Lys482 (Figure 5A). The other three phenolic acid esters had similar binding modes (Supplementary Figures S3–S5). For the most suitable and largest substrate, the oxygen atom of the propyl group in tannic acid, regarded as hydrogen bond donor, forms hydrogen bonds with Gly485, Glu492, Gly493, Thr498, and Asn506. In addition, the two oxygen atoms of the phenolic hydroxyl group in tannic acid form two hydrogen bonds with the oxygen atom of the side chain of Asn483. VDW interactions were also formed between ethyl gallate and the surrounding residues (Figure 5B). These docking simulations may provide insights into the substrate specificity of TanA_Lb_, although the exact enzyme structures were not obtained (Wang et al., 2020).

**TABLE 3:** The docking score of ligands with TanA_Lb_.

Receptor	Ligand	Docking score (Kcal/mol)
TanA_Lb_	EG	−4.38
TanA_Lb_	MG	−4.19
TanA_Lb_	PG	−4.64
TanA_Lb_	LG	−5.58
TanA_Lb_	TA	−11.39

**FIGURE 6:**
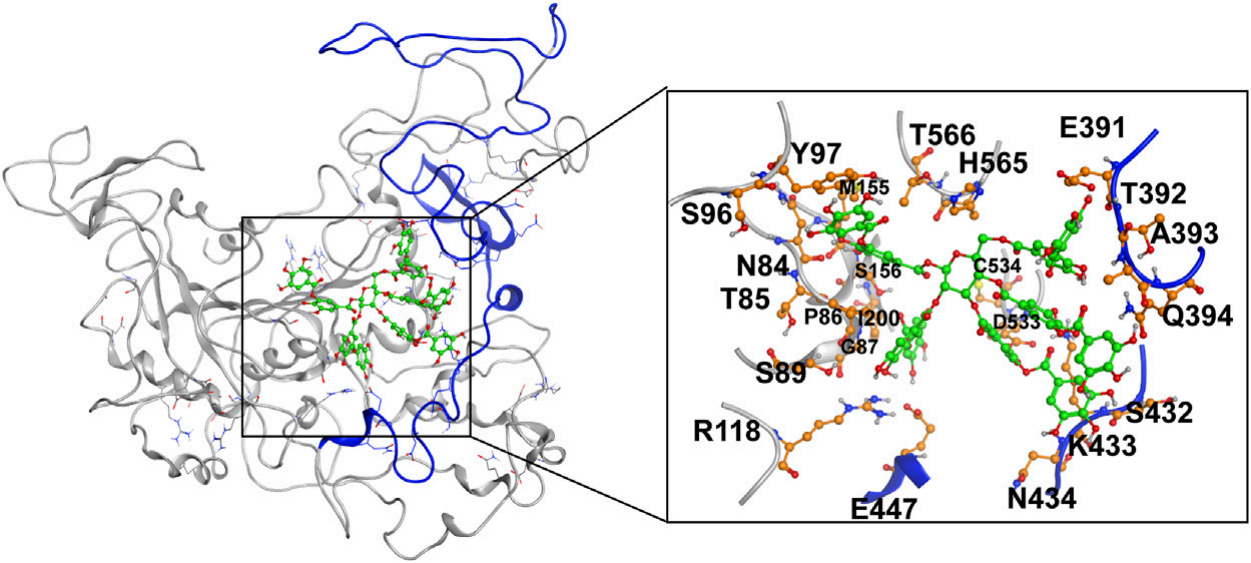
The final stable structure model after 500-ns molecular dynamics (MD) simulation. The sequence from 341 to 450 is highlighted with blue color. The atoms in tannic acid in binding area are depicted as green, and the residues in TanA_Lb_ involved in binding are depicted as orange.

**FIGURE 7:**
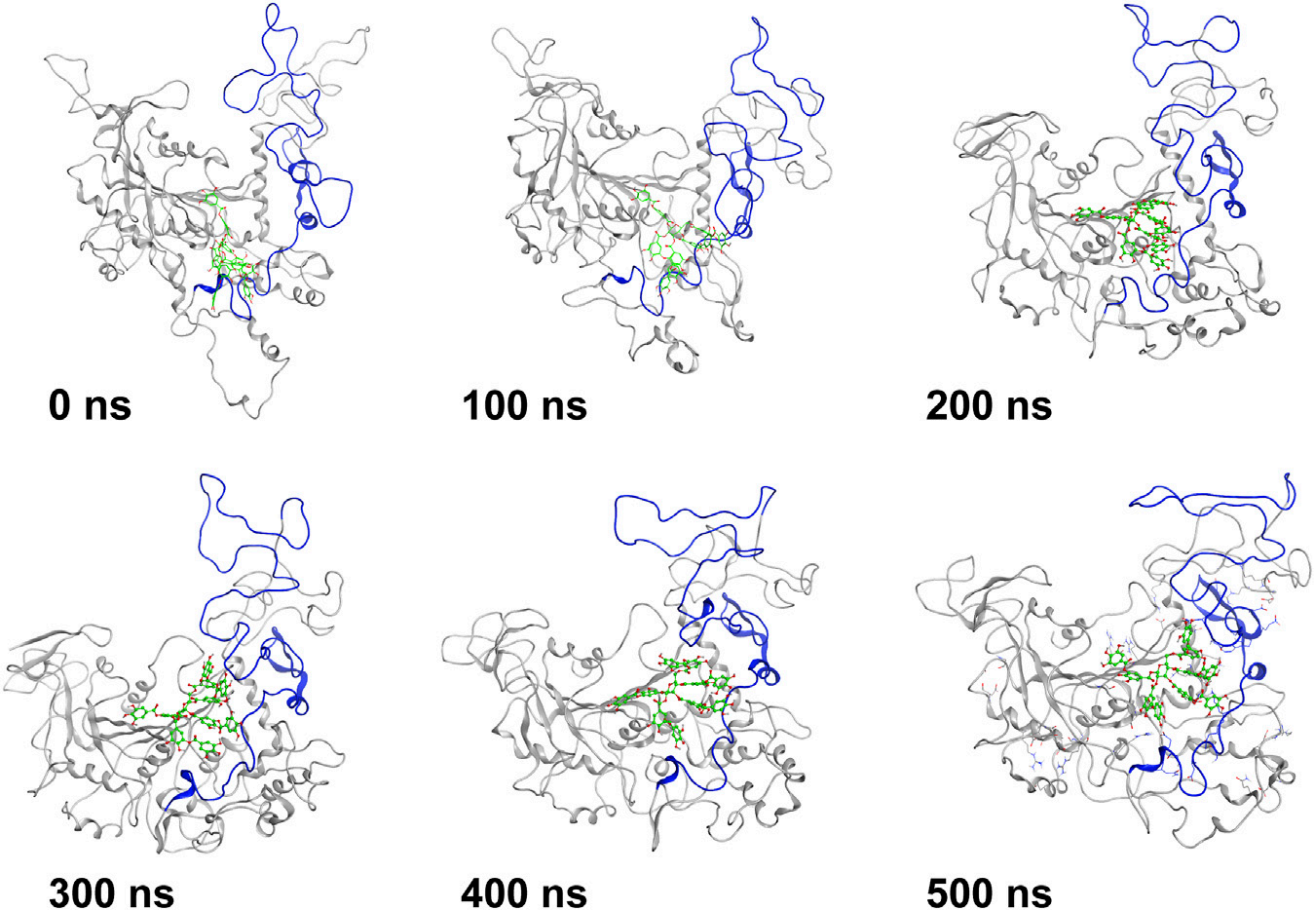
The structures at different simulation times of the complex. Residues 341–450 are shown as blue ribbons, and tannic acid is shown as green atoms. Other residues are shown as gray ribbons.

**FIGURE 8:**
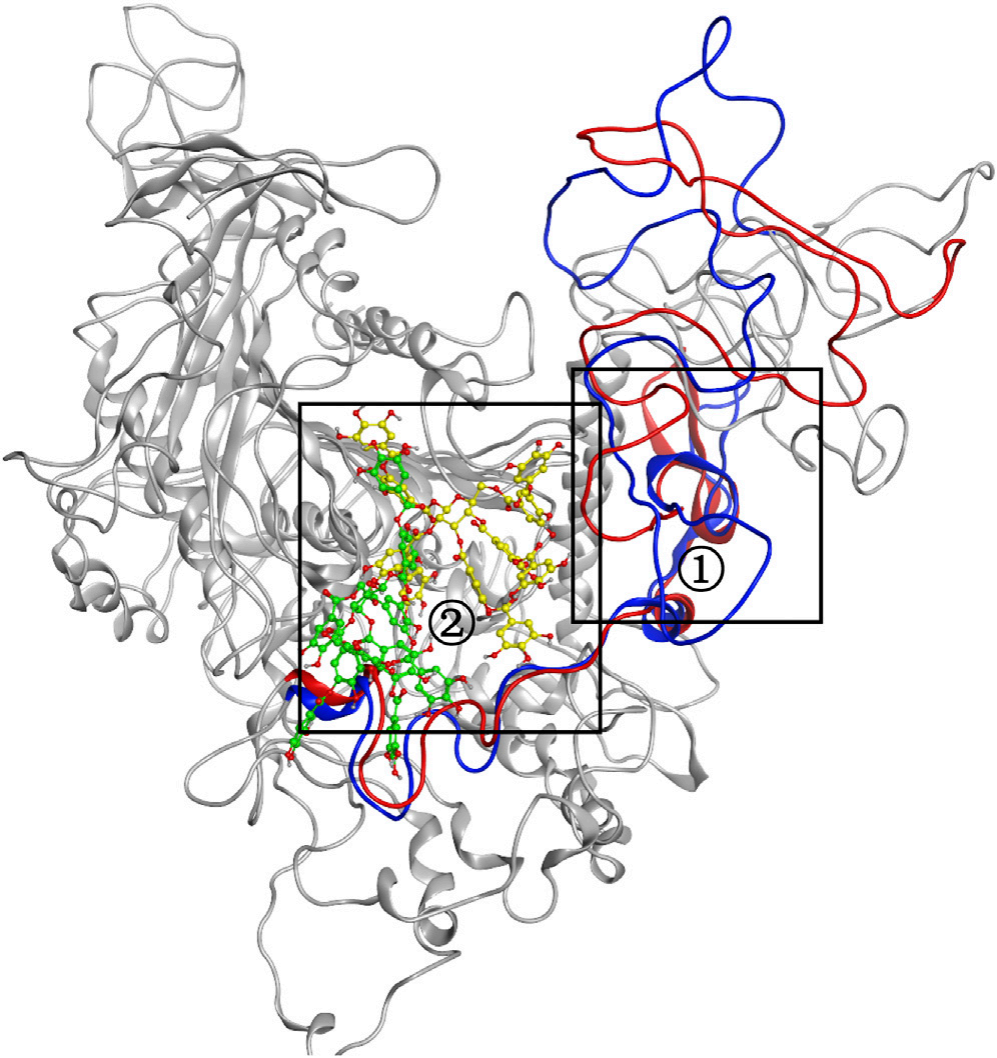
The conformational change for the complex of TanA_Lb_ and tannic acid. Loops 341–450 of 0 -s simulation structure are shown in blue, and the ligand is shown in green. Meanwhile, loops 341–450 of 500-ns simulation structure are shown in red, and the ligand is shown in yellow.

MD simulation was carried out to explore the role of the insertion part (residue 341–450). The results showed that the root means square deviation (RMSD) of heavy atoms of TanA_Lb_ was less than 10.0 Å, that of tannic acid was less than 5.0 Å, and the system achieved equilibrium within 150 ns (Supplementary Figure S6). The final stable 3D structure binding model of the complex of TanA_Lb_ and tannic acid is shown in Figure 6 and the binding energy of key residues are listed in Supplementary Tables S1, S2. During the MD simulation, tannic acid tended to approach the target loop structure (residue 341–450), and the other loop (residue 389–395) moved toward tannic acid to form a stable interaction (Figure 7). The main conformational change was the loop from amino acid 389 to 395, which swung like a pair of hands holding tannic acid. The remaining parts, which mainly interact with tannic acid, were the loop structures of 431–435 and 446–448, which were relatively stable here (Figure 8). It is supposed that the loop (residue 341–450) is a flap-like domain working for binding bulk substrates such as tannic acid, like the flexible structure in esterases (Truongvan et al., 2016).

## Conclusion

A novel bacterial tannase from a species of *Lachnospiraceae* has been cloned and overexpressed in *E. coli* BL21 (DE3), purified, and characterized. It exhibited maximal activity at pH 7.0 and 50°C, and retained more than 70% relative activity from 30°C to 55°C. The enzyme retained more than 80% of the initial activity after incubation for 2 h in the pH range 6.5–7.5. TanA_Lb_ is stable in the presence of surfactants such as SDS, DSMO, and TritonX-100. TanA_Lb_ has broad substrate specificity, and investigating the detailed kinetic characteristics of TanA_Lb_ advances our knowledge about this bacterial tannase at the molecular level. MD simulation revealed an assumed flap-like lid, which was beneficial to the interactions with the substrates. This may be helpful to guide the application of tannase and provide the theoretical basis for the modification of tannase. As a novel bacterial tannase, TanA_Lb_ has demonstrated superior properties that may help improve the industrial biodegradation or bioconversion of tannins. Moreover, TanA_Lb_ is from *Lachnospiraceae bacterium* in ruminant gastrointestinal tract, and the research probably helps to increase our understanding of the relationship between tannases and gut microbiota and health.

## Data Availability

The original contributions presented in the study are included in the article/Supplementary Material. Further inquiries can be directed to the corresponding authors.
