# Supplementary material for: Biochemical and Structural Characterization of a Novel Bacterial Tannase From Lachnospiraceae bacterium in Ruminant Gastrointestinal Tract
Source: Front Bioeng Biotechnol. 2021 Dec 15;9:806788. doi: 10.3389/fbioe.2021.806788 (PMC8715002; doi:10.3389/fbioe.2021.806788)
Supplement: Supplementary file 1 [file DataSheet1.pdf]

Supplementary Data

Table S1 Average Binding Energy and its Components Obtained from the MM-GBSA Calculation for the complex

**MM-GBSA**	ΔE_{vdw}	ΔE_{ele}	ΔG_{polar}	$\Delta G_{nonpolar}$	ΔG_{total}
	-62.84±6.23	-140.30±21.77	190.58±19.27	-11.62±0.79	-24.18±5.92

Table S2 Binding energy and its components of key residues

**Residue**	ΔE_{vdw}	ΔE_{ele}	ΔG_{polar}	$\Delta G_{nonpolar}$	ΔG_{total}
ASN84	0.2965	-3.1678	1.2319	-0.0701	-1.7095
THR85	-0.5293	0.5934	-0.5462	-0.0005	-0.4826
PRO86	-3.8054	-1.9612	1.8743	-0.5482	-4.4405
GLY87	-1.3069	-1.0802	1.1456	-0.1029	-1.3444
TYR88	-0.2625	-0.1332	0.212	-0.0006	-0.1843
SER89	-0.3867	-1.0197	1.2547	-0.1045	-0.2563
TYR97	-3.0746	-0.2836	1.2382	-0.4914	-2.6113
GLY154	-0.0319	-0.0072	-0.1764	0	-0.2155
MET155	-0.9664	-0.8337	0.291	-0.046	-1.5552
SER156	-1.0177	-1.1814	1.4453	-0.1718	-0.9256
GLY158	-0.0523	-0.2072	0.0661	0	-0.1935
TRP197	-0.1759	0.4168	-0.5449	-0.0047	-0.3087

ILE200	-1.1424	-0.0048	0.0041	-0.2712	-1.4143
TYR390	-0.9825	-0.1923	0.9932	-0.2047	-0.3863
GLU391	-1.0626	-8.7716	8.5871	-0.4094	-1.6564
THR392	-1.9668	-1.9991	2.4646	-0.1554	-1.6567
ALA393	-2.1521	-1.818	1.958	-0.4675	-2.4795
GLN394	-0.8574	-2.8423	2.9183	-0.2748	-1.0562
TYR396	-0.2975	-0.1062	0.3122	-0.0169	-0.1085
SER432	-1.0985	-0.5683	1.8381	-0.3213	-0.15
LYS433	-1.8658	2.1092	-0.7608	-0.3484	-0.8658
ASN434	-0.4918	-0.4205	0.7635	-0.1077	-0.2565
PHE438	-0.2062	-0.0284	0.1417	-0.0725	-0.1655
GLN445	-0.7043	-0.4355	0.939	-0.1402	-0.341
GLY446	-0.2096	0.1592	-0.1443	-0.0316	-0.2263
GLU447	0.6079	-20.7081	16.7905	-0.3654	-3.6751
GLN531	-0.1697	-0.2375	0.2147	-0.0006	-0.193
ASP533	1.9391	-25.0192	18.592	-0.425	-4.913
HIE565	-2.6153	-0.6375	1.3067	-0.5222	-2.4683
THR566	-1.3792	-0.161	0.4575	-0.1836	-1.2663

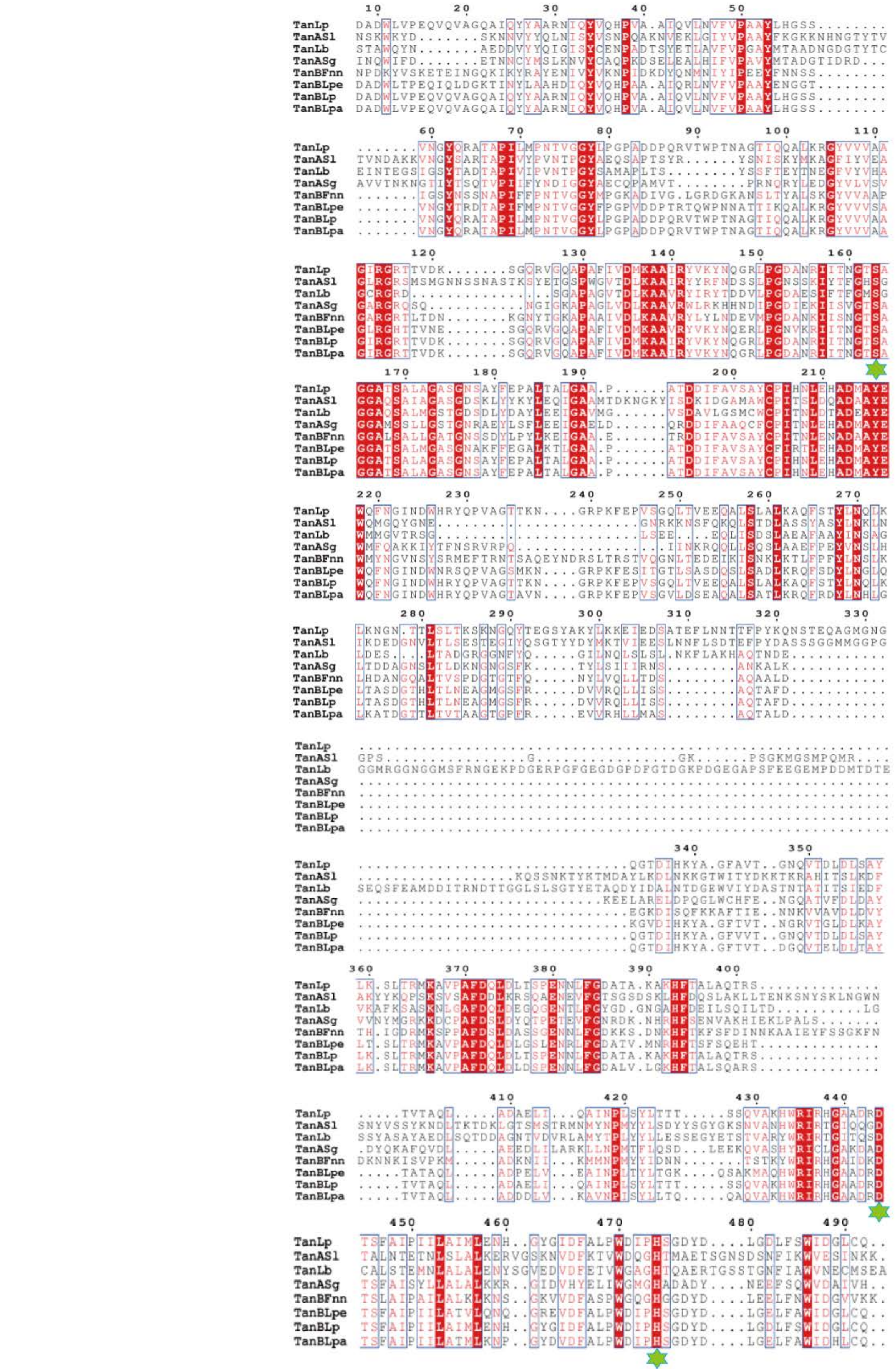

Fig.S1 Multiple sequence alignment of $TanA_{Lb}$ from *Lachnospiraceae* (MBQ6323131.1), $TanA_{Lp}$ from *Lactiplantibacillus plantarum* (BAH20446.1), $TanA_{Sl}$ from *Staphylococcus lugdunensis* (BAF03594.1), $TanA_{Sg}$ from *Streptococcus gallolyticus* UCN34 (YP_003431024.1), $TanB_{Fnn}$ from *Fusobacterium nucleatum subsp. polymorphum* (ALQ42581.1), $TanB_{Lpe}$ from *Lactiplantibacillus pentosus* (BAN10247.1), $TanB_{Lp}$ from *Lactobacillus plantarum* (BAG68453.1) and $TanB_{Lpa}$ from *Lactiplantibacillus paraplantarum* (BAN10246.1). The three conserved catalytic residues Ser, Asp, and His are indicated by green stars.

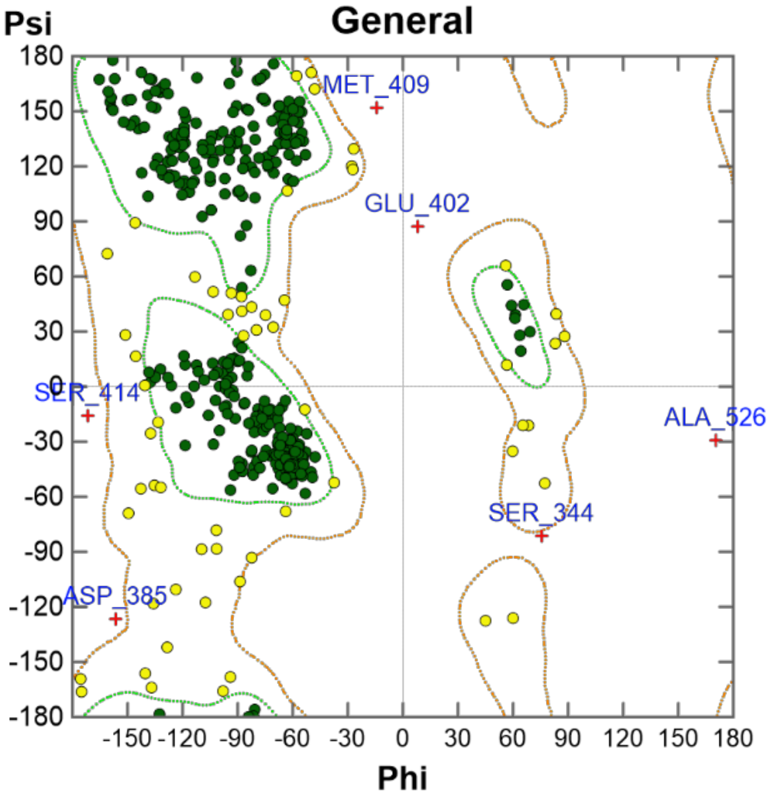

Fig.S2 Ramachandran plot for $TanA_{Lb}$. Dark green dots represent the residues in favored regions; yellow dots represent the residues in allowed regions, red cross represent the residues in irrational regions.

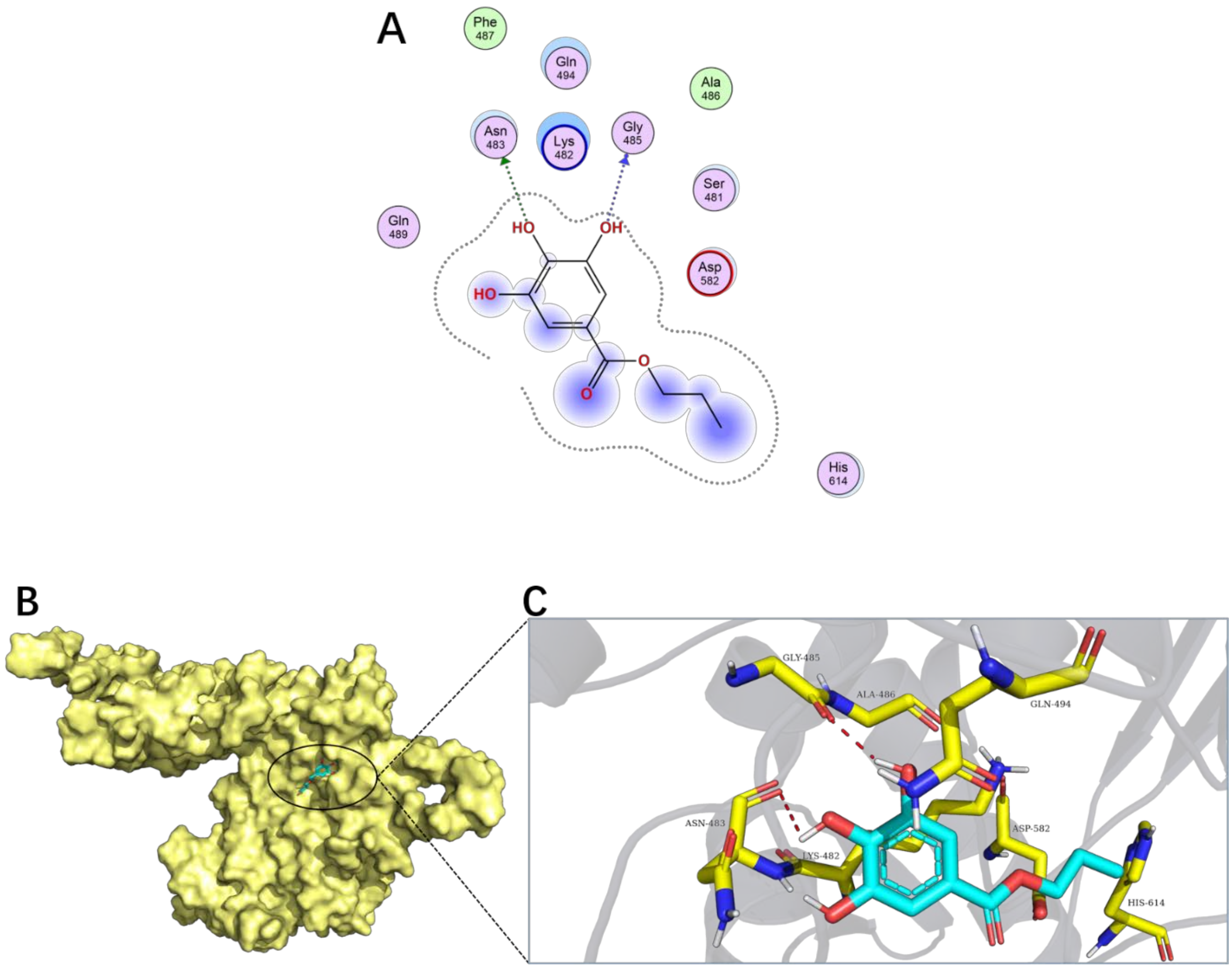

Fig. S3 Complex of $TanA_{Lb}$ and propyl gallate. (A) The 2D binding mode of $TanA_{Lb}$ and propyl gallate. (B) The binding model of propyl gallate on molecular surface of $TanA_{Lb}$. Propyl gallate is colored in cyan, the molecular surface of tannase is colored in pale yellow. (C) The 3D binding mode of tannase and propyl gallate. Propyl gallate is colored in cyan, the surrounding residues in the binding pockets are colored in yellow, the backbone of the receptor is depicted as white cartoon with transparency.

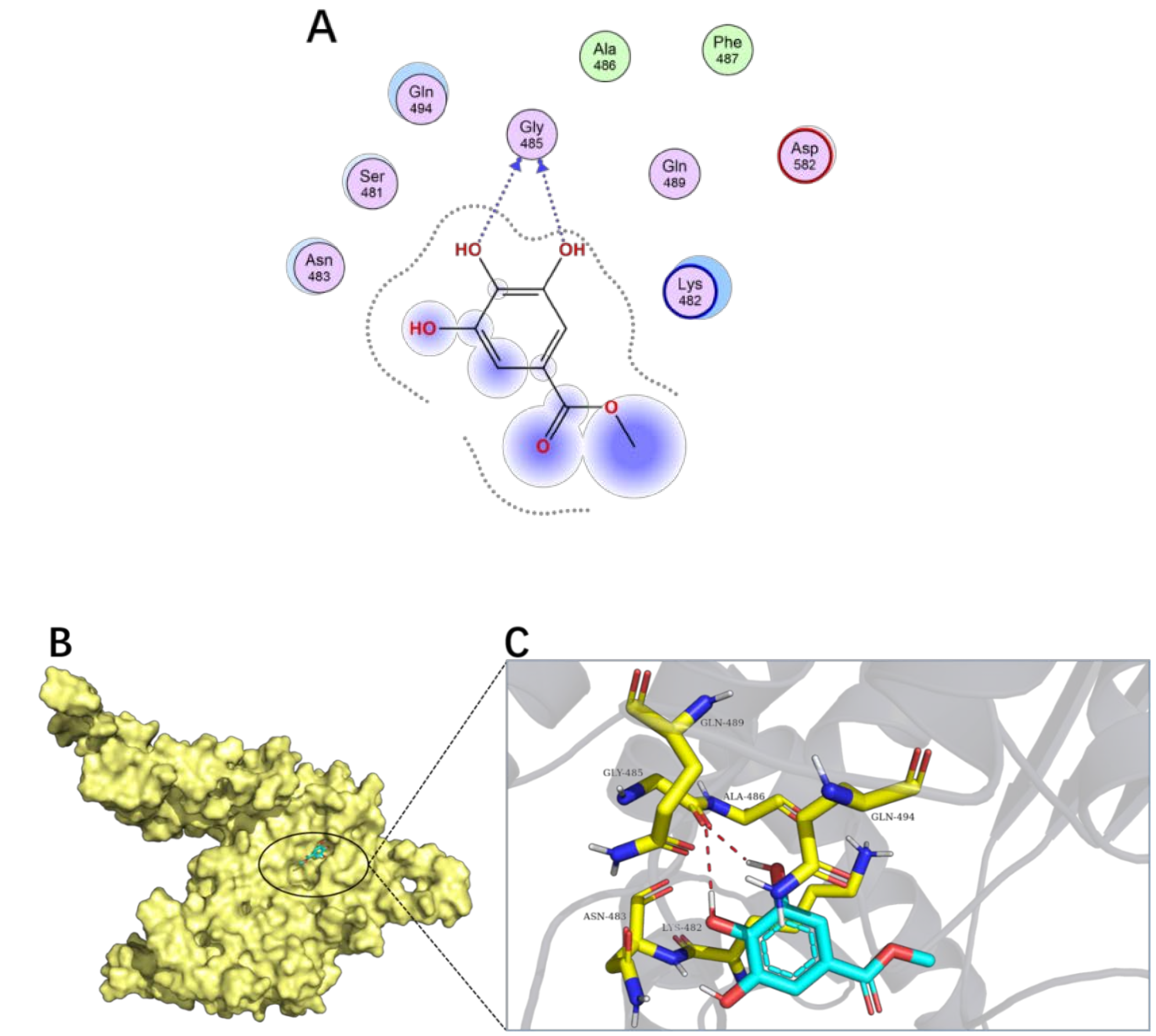

Fig. S4 Complex of TanA$_{Lb}$ and methyl gallate. (A) The 2D binding mode of TanA$_{Lb}$ and methyl gallate. (B) The binding model of methyl gallate on molecular surface of TanA$_{Lb}$. Methyl gallate is colored in cyan, the molecular surface of TanA$_{Lb}$ is colored in pale yellow. (C) The 3D binding mode of TanA$_{Lb}$ and methyl gallate. Methyl gallate is colored in cyan, the surrounding residues in the binding pockets are colored in yellow, the backbone of the receptor is depicted as white cartoon with transparency.

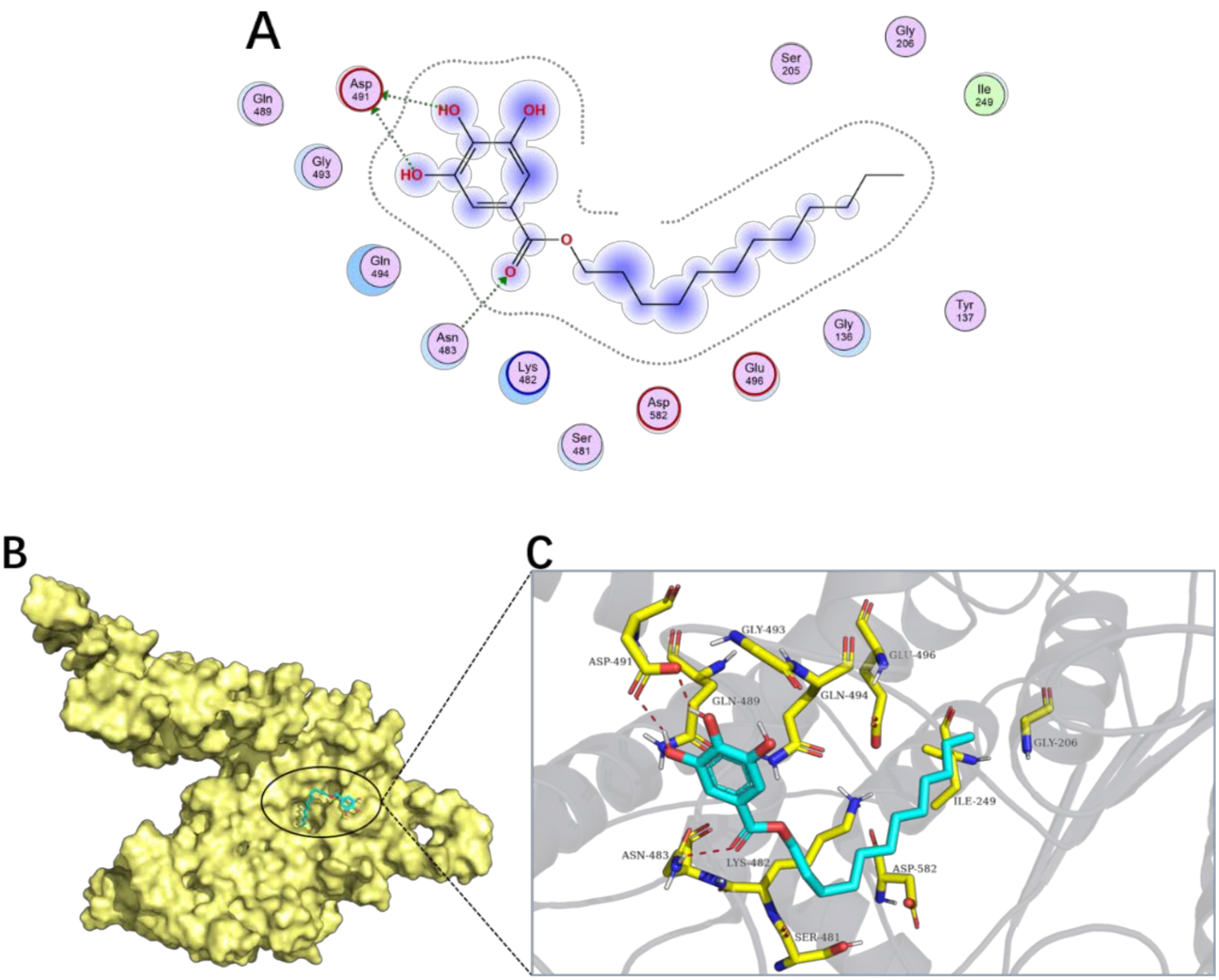

Fig. S5 Complex of $TanA_{Lb}$ and Lauryl gallate. (A) The 2D binding mode of $TanA_{Lb}$ and lauryl gallate. (B) The binding model of lauryl gallate on molecular surface of tannase. Lauryl gallate is colored in cyan, the molecular surface of $TanA_{Lb}$ is colored in pale yellow. (C) The 3D binding mode of $TanA_{Lb}$ and Lauryl gallate. Lauryl gallate is colored in cyan, the surrounding residues in the binding pockets are colored in yellow, the backbone of the receptor is depicted as white cartoon with transparency.

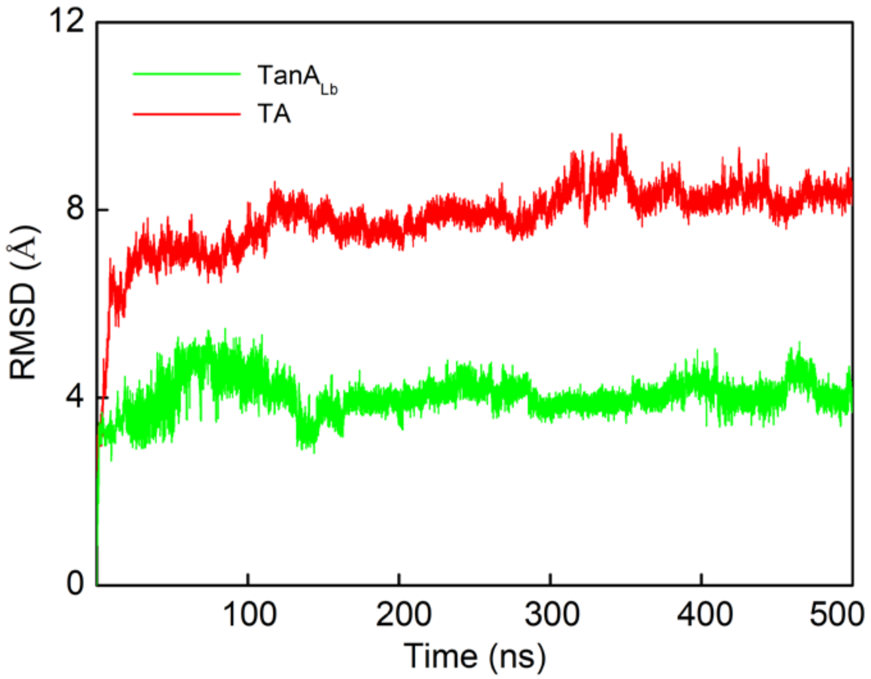

Fig. S6 RMSD of the TanA$_{Lb}$ and tannic acid during the 500ns simulation.
